# Dynamic, Not Isometric Resistance Training Improves Muscle Inflammation, Oxidative Stress and Hypertrophy in Rats

**DOI:** 10.3389/fphys.2019.00004

**Published:** 2019-01-22

**Authors:** Rodrigo Vanerson Passos Neves, Thiago Santos Rosa, Michel Kendy Souza, Alexsander José Costa Oliveira, Gustavo Neves Souza Gomes, Bernardo Brixi, Luiz Humberto Rodrigues Souza, Lysleine Alves Deus, Herbert Gustavo Simões, Whitley Jo Stone, Jonato Prestes, Milton Rocha Moraes

**Affiliations:** ^1^Graduate Program in Physical Education, Catholic University of Brasília, Brasília, Brazil; ^2^Graduate Program in Exercise Physiology, Ibirapuera University, São Paulo, Brazil; ^3^Physical Education School, University Center Estácio, Brasília, Brazil; ^4^Physical Education, State of Bahia University (UNEB) – DEDC/XII Campus, Guanambi, Brazil; ^5^School of Nutrition, Kinesiology, and Psychological Sciences, University of Central Missouri, Warrensburg, MO, United States

**Keywords:** muscle strength, strength training, static resistance training, blood glucose, inflammation, cytokines, antioxidant defense, oxidative stress

## Abstract

This study aimed to compare the effects of dynamic (DRT) and isometric (IRT) resistance training on blood glucose, muscle redox capacity, inflammatory state, and muscle strength and hypertrophy. Fifteen 12-week-old male Wistar rats were randomly allocated into three groups: control group (CTL), DRT, and IRT, *n* = 5 animals per group. The animals were submitted to a maximal weight carried (MWC; every 15 days) and maximum isometric resistance (MIR; pre- and post-training) tests. Both training protocols were performed five times a week during 12 weeks, consisting of one set of eight uninterrupted climbs for 1 min with a 30% overload of MWC. The animals in the IRT group remained under isometry for 1 min. The DRT group experienced greater MWC from pre- to post-training compared to the CTL and IRT groups (*p* < 0.0001). The DRT and IRT groups displayed similar gains in MIR (*p* = 0.3658). The DRT group exhibited improved glycemic homeostasis (*p* = 0.0111), redox (*p* < 0.0001), and inflammatory (*p* < 0.0001) balance as compared with CTL and IRT groups. In addition, the improved glycemic profile was associated with an increase in muscle strength and hypertrophy, improvement in redox balance and inflammation status. We conclude that DRT was more effective than IRT on increasing cross-sectional area, but not muscle strength, in parallel to improved blood glucose, inflammatory status, and redox balance.

## Introduction

Dynamic resistance training (DRT) is widely prescribed as an adjuvant in the treatment and prevention of cardiometabolic diseases (Church et al., 2010; Moraes et al., 2012; Nunes et al., 2016). The benefits of DRT are primarily due to the increase in muscle strength and hypertrophy (Dudley, 1988; Kraemer et al., 1996). Impairments in muscle function are intimately related with metabolic disorders (Yang, 2014), sedentary lifestyle, obesity, cardiovascular diseases, as well as the aging process, and are negatively correlated with muscle power and strength (Artero et al., 2011; Yang, 2014; Pion et al., 2017).

Nevertheless, isometric resistance training (IRT) has received special attention and praise when implementing in rehabilitation programs in osteoarthritis (Lambova, 2017). However, the effects of IRT on metabolic parameters have been minimally investigated. A previous study demonstrated that acute isometric resistance exercise *in situ* increased glucose uptake through the translocation of GLUT-4 via IGF-1 pathway (Kido et al., 2016). One of the first studies to investigate the chronic effects of IRT on glucose metabolism revealed a reduction of glucose intolerance in experimental model (Kruger et al., 2013). The study also reported an increase in the GLUT-4 gene expression in muscle tissue, but the authors were unable to explain the mechanisms responsible for the benefits. However, it is unlikely that the low caloric expenditure involved in isometric exercise would improves glycemic homeostasis.

Oxidative stress, inflammation, and nitric oxide (NO) bioavailability are involved in glucose uptake by the muscle (Etgen et al., 1997; Xie et al., 2017). NO has an important role in glucose uptake signaling, wherein the decrease in NO bioavailability is associated with insulin resistance in myocytes (Etgen et al., 1997; Carvalho-Filho et al., 2005). Alternatively, muscle contraction itself chronically increases NO bioavailability (Guizoni et al., 2016) and stimulates the uptake of glucose independent of insulin through several mechanisms not fully understood (Sylow et al., 2017). Attenuated inflammation induced by exercise is another factor responsible for increasing insulin sensitivity in skeletal muscle (Da Silva et al., 2010). Evidence exists supporting DRT in the reduction of oxidative stress and inflammation in humans (Padilha et al., 2015) and rodents (Padilha et al., 2017). While indirect, the hypertrophic qualities of DRT would promote greater glucose uptake simply by amplifying cross-sectional area (CSA) (Yang, 2014). The present literature is less clear regarding the influence of IRT on these metabolic parameters. Hence, it remains unknown if isometric muscle actions promotes long-term glycemic improvements when compared to a sedentary lifestyle. Some studies have suggested that acute isometric exercises increases the uptake of glucose by muscle tissue (Ahlborg et al., 1972; Peltoniemi et al., 2001). However, it remains unknown if such improvement is sufficient to alter long-term glycaemia. Thus, we designed this experimental to test if IRT improves the glycaemia when compared to DRT and a sedentary lifestyle.

Therefore, the present study aimed to compare the effects of DRT and IRT on glycemic homeostasis, muscle redox and inflammatory states, along with muscle strength and hypertrophy in Wistar rats. We hypothesized that DRT would promote improvements in glycaemia, inflammatory, and redox profiles, as well as improved hypertrophy compared to IRT and sedentary rats.

## Materials and Methods

### Animals

Twelve-week-old male Wistar rats were obtained from the laboratory of the Basic Processes in Psychology of the Catholic University of Brasília. The animals were randomized into three groups with five animals per group: control group (CTL), DRT, and IRT. Body mass values 378 ± 20 g (CTL), 368 ± 26 g (DRT) and 348 ± 40 g (IRT) and fasting glycaemia 100 ± 4 mg/dL (CTL), 97 ± 6 mg/dL (DRT), and 100 ± 7 mg/dL (IRT) were collected at baseline. This study was approved by the Committee on Ethics in the Use of Animals (CEUA/UCB) registered under No. 004/16. The study was conducted according to the guidelines of the National Council of Control of Animal Experimentation (CONCEA). Each group of rats were kept in the laboratory of the Physical Education and Health Studies (LEEFS), with a light-dark cycle of 12/12 h in plastic enclosures (*n* = 5 per enclosure) at a temperature of 22 ± 2°C and relative humidity of 55 ± 10%. Animals were fed with a standard rat chow diet (Nuvital^®^ CR1, São Paulo, Brazil) and granted water *ad libitum*.

### Adaptation to the Ladder Climbing Training Apparatus

In order to reduce the levels of stress in this new task, all rats were conditioned to the resistance training protocol by climbing a vertical ladder (110 cm high × 18 cm wide × 2 cm high, 80° inclination). A housing chamber (L × W × H = 20 cm × 20 cm × 20 cm) located at the top of the ladder was used as a shelter for the animals during the resting period. Familiarization to the apparatus consisted of climbing the ladder without load, three times per week for 2 weeks on non-consecutive days, totalizing six sessions of familiarization. This protocol was first described by Neves et al., 2016. Forty-eight hours after familiarization, all rats were submitted to the maximal weight carried (MWC) test.

### Maximal Weight Carried Test

The MWC consisted of 4–9 ladder climbs with progressive incremental loads. For the initial climb, the load carried was 75% of the animal’s body weight. Then an additional 30 g of overload was added until a maximum load was reached, identified as the animal’s failure. Failure was determined when the animal failed to climb up the ladder, despite three consecutive stimuli to the tail (using tweezers). A rest period of 60 s was used between each climb. The heaviest load performed successfully along the entire length of the ladder was considered the MWC. The subsequent test session consisted of a ladder climb with 50, 75, 90, and 100% of the rat’s previous MWC with a rest interval of 60 s between each climb. After that, a load of 30 g was added until a new MWC was determined; the recovery period between each overloaded climb was 120 s (Neves et al., 2016). This procedure was performed every 15 days to adjust the load of both training interventions (DRT and IRT) and to investigate the behavior of muscle strength in all animals throughout the experimental protocol.

### Familiarization to the Isometric Resistance Training

**FIGURE 1:**
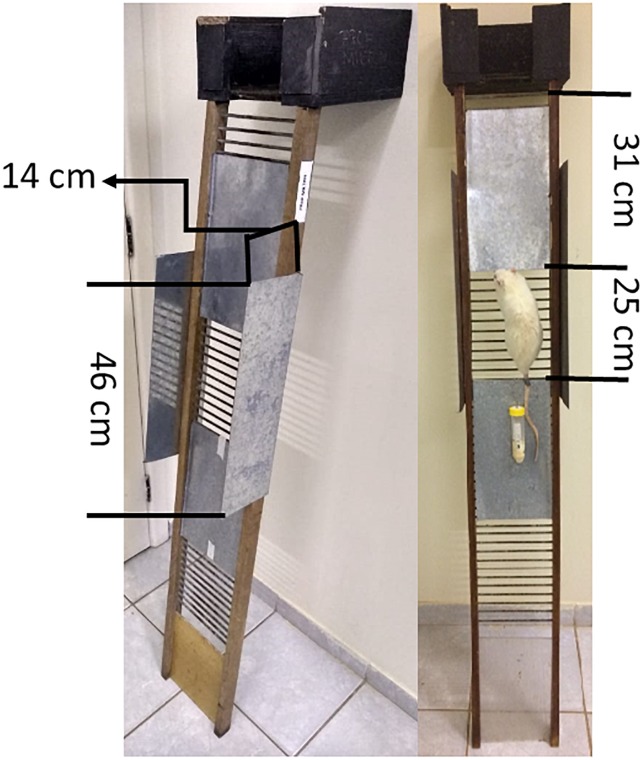
Apparatus for isometric resistance training in rats.

Two days after MWC evaluation, the rats were adapted to the IRT. All rats were conditioned on the same ladder described before. Two metal plates were fixed on the ladder with a space of ∼25 cm between them. This model was developed with the purpose to preclude movements (i.e., climbing the ladder) by the rat on the ladder, thereby creating a 100% isometric system (Figure 1). The familiarization to the isometric exercise was implemented for six consecutive days. On the first day, all animals were positioned in this space (∼25 cm) between the plates for 10 s of isometry. Each sequential session deployed and additional 10 s of exercise until achieving 60 s of isometry (marked during session six). Thereafter, the animals were exposed to a progressive load of 5–30% of the MWC over six consecutive sessions (1 week). Rats started with 5% of the MWC on day 1, and for each new session, an additional 5% MWC was accumulated up to 30% of MWC by the sixth session. The overload was fixed to the proximal part of the animal’s tail with a Coastlock Snap Swivel and Scotch 23 Rubber Tape (Scotch 3M, São Paulo, Brazil), as described by Neves et al., 2016.

### Maximal Isometric Resistance Test

Forty-eight hours after the familiarization to the isometric resistance training, all rats were placed on the ladder in the space between the metal plates for the maximal isometric resistance (MIR) test. The test consisted of placing the rats in the space between the metal plates of ∼25 cm as long as possible. The time was measured until their paws’ grip failed leading to their fall on a cushion similar to the procedure described by Kruger et al., 2013. This MIR test was assessed before and after 12 weeks of training (DRT, IRT, and CTL).

### Glucose Tolerance Test (GTT)

The GTT was performed at the baseline (forty-eighty hours after the first MIR test) and post-training (forty-eighty hours after the last MIR test). A small incision was made at the tip of the animal’s tail to collect blood samples after 6–8 h of fasting using a portable glucometer (Accu-Chek Advantage; Roche Diagnostics Co., Indianapolis, IN, United States). This blood collection determined baseline and served the glucose dosage for the GTT and served as time zero (t0). After baseline collection, 2 g of 80% D-glucose solution per kilogram of body weight was injected intraperitoneally, thereafter blood glucose concentrations were measured after 15, 30, 60, 90, and 120 min, respectively. Glucose intolerance was assessed using the area under the curve (AUC) of the GTT (Ouwens et al., 2005).

### Dynamic and Isometric Resistance Training Protocols

Two days after the GTT both resistance training protocols started. DRT was performed five times per week for 12 weeks, lasting an average of 22 min per session. Sessions consisted of 8, 1 min sets with an overload of 30% MWC. The size of the ladder required the rats to perform 8–12 dynamic movements per climb with 2 min rests between sets. The apparatus DRT for rats was shown by Neves et al., 2016.

The IRT group performed the training with the same exercise prescription as the DRT group. The only exception was that the animals of this group performed the exercise in isometry as described in the MIR test. Initially, a pilot study imposing loads from 5 to 70% of MWC indicated that 30% was the maximum overload the rats could isometrically sustain for more than 60 s. Coincidentally, is the same load applied in studies with humans on hemodynamic outcomes (Millar et al., 2014; Souza M.K. et al., 2018). The rats of CTL group did not perform any type of training beyond the physical tests (e.g., MWC and MIR). Additionally, body weight and food intake were evaluated weekly in all animals.

### Euthanasia

**FIGURE 2:**
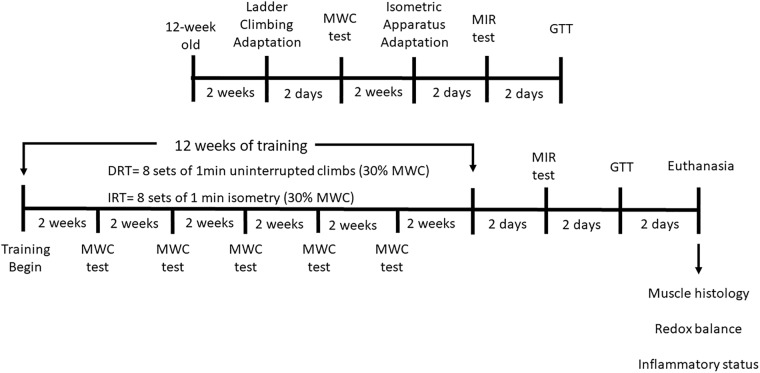
Experimental design. DRT, dynamic resistance training; IRT, isometric resistance training; MWC, maximal weight carried; MIR, maximal isometric resistance; GTT, glucose tolerance test. Male Wistar rats were selected at 12 weeks old. All rats were submitted to familiarization with dynamic strength exercise for 2 weeks. Two-days after, the animals were submitted to the maximal weight carried (MWC) test. Afterward, the animals were adapted to the isometric exercise for 2 weeks. Subsequently, the MIR and GTT tests were carried out, both tests were applied with 2 days of difference between them. Then, the rats of the DRT and IRT groups trained for 12 weeks, each one with their respective muscular action, dynamic and isometric, respectively. Muscle strength (MWC) was measured every 2 weeks throughout the training protocol, in order to evaluate the muscular strength behavior and to adjust the training load. After the end of the training, the MIR and GTT tests were applied with 2 days of difference between them. Euthanasia was performed after 2 days, the quadriceps and tibialis anterior muscles were collected for evaluation of the transverse section. The parameters of the redox and inflammatory state were measured in the tibilialis anterior muscle.

Two days after the last GTT, the animals were anesthetized using Ketamine (80 mg/kg IP) and Xylazine (10 mg/kg IP), followed by cardiac puncture exsanguination. The tibialis anterior muscle (right paw) was first stored at -80°C for the further redox and inflammatory analysis. Analyzes of redox status and inflammatory profile were performed only in the tibialis anterior muscle. The quadriceps and tibialis anterior muscles were collected and weighed. For the histological analysis the left paw muscles were collected. The experimental design of all phases of the research is illustrated in Figure 2.

### Histomorphometric Analysis

After collection, the muscles were rapidly dissected to separate connective tissues and fat, and then dipped into 10% formaldehyde (10 mM buffer-phosphate, pH 7.4) for further histological paraffin processes. Tissues were embedded in paraffin blocks, sliced at 5 and 6 μm, and the CSA of the muscle was measured in the medial portion after staining with Hematoxylin-eosin (Tang et al., 2014). Photomicrographs were obtained using a light microscope (Leica DM1000, Wetzlar, Germany, 20 × objective and 10 × ocular). For the analysis of muscular cross section, 20 images per animal were obtained, totaling 100 images per group. An area of 10 cells per photomicrograph was measured. To analyze the area of the cells, the AxioVision Rel, 4.8 software was used (Carl Zeiss, IL, United States).

### Muscles Preparation

The tibialis anterior muscle was thawed, excised and transferred to ice-cold containers with 0.9% NaCl and homogenized in 0.1 mol/L Tris-HCl buffer (pH 7.4). After of the homogenization process were analyzed the NO, lipid peroxidation, total antioxidant capacity, superoxide dismutase (SOD) and catalase activities, glutathione (GSH) and glutathione disulfide (GSSG), c-reactive protein (CRP), tumor necrosis factor-α (TNF-α), interleukin-4 (IL-4) and interleukin-10 (IL-10). The protein content was analyzed by Bradford assay (Stocks et al., 1974).

### Muscular Nitric Oxide

The NO was measured using the Griess reaction (Miranda et al., 2001), according to the following protocol: homogenate of muscle tissue samples were deproteinized with zinc sulfate (20%) in PBS; 100 μL of each sample were disposed in duplicates in a 96-well plate, a solution of 100 μL of vanadium chloride, 50 μL sulfanilamide and 50 μL of *N*-(1-Naphthyl) ethylenediamine dihydrochloride, were added, an standard nitrite curve were also added. After that, the plate was homogenized and incubated for 40 min at 37°C. The samples were read in spectrophotometer at 540 nm.

### Lipid Peroxidation

The formation of thiobarbituric acid-reactive substances (TBARS) is one the most used methods to assess the final products of lipid peroxidation and estimates the oxidative damage in cells and tissues (Gutteridge, 1995). Basically, the homogenate muscle samples were diluted in 320 μL MiliQ H_2_O (1:5) and 1 mL of trichloroacetic acid (TCA) 17.5%, pH 2.0 was added, following the addition of 1 mL of thiobarbituric acid (TBA) 0.6%, pH 2.0. After the homogenization, the samples were kept in a water bath for 30 min at 95°C. Then, samples were immersed in ice and 1 mL of TCA 70%, pH 2.0 were added, and another incubation of 20 min at room temperature were done. Afterward, the samples were centrifuged (3000 g for 15 min) and the supernatant were read in a spectrophotometer at 540 nm. The concentration of lipid peroxidation products were calculated using the molar extinction coefficient equivalent to malondialdehyde (MDA-equivalent = 1.56 × 10^5^ M^-1^cm^-1^). The protocol was adapted from Ohkawa et al., 1979.

### Redox Capacity Parameters

The antioxidant chemicals were measured using commercial kits following the manufacturer’s protocol. The SOD activity was measured using the SOD assay kit (Sigma-Aldrich^®^, CA, United States), read at 450 nm. Total antioxidant capacity was measured using the QuantiChrom antioxidant assay kit (QuantiChrom BioAssay Systems, CA, United States), which measures the Trolox-equivalent antioxidant capacity. Catalase activity was measured using the Amplex^TM^ Red catalase assay kit (Thermo Fisher Scientific^®^, MA, United States) at 560 nm. The GSH and GSSG were measured in a spectrophotometer at 412 nm using the Glutathione Assay Kit (Sigma-Aldrich^®^, CA, United States) (Akerboom and Sies, 1981).

### Inflammatory Status

The pro-inflammatory (CRP and TNF-α), anti-inflammatory (IL-4 and IL-10) cytokines were determined by enzyme-linked immunosorbent assay with Cytokines ELISA kits (R&D Systems, Inc., United States). All samples were run as duplicates and the mean value was reported. The reproducibility of the cytokine dosages was verified by Pearson’s coefficient of variation, and varied between 10 and 15% of the data, which presented a good reliability. Assay sensitivity for cytokines was 5.0 pg ml^-1^ in the range is up to 1000 ng l^-1^ for all measured parameters. The muscle levels of CRP, TNF-α, IL-4, and IL-10 were analyzed according to the manufacturer’s instructions.

### Statistical Analysis

Initially, was calculated the statistical power for F test types such as ANOVA, which we found for a large effect size, with an α error of 0.05, power (1–β error) of 80%, total sample size 15. This analysis was performed at Gpower^®^. Data are presented as the mean ± standard deviation. Normality and homoscedasticity were assessed using the Shapiro-Wilk and Levene tests, respectively. A repeated measures two-way ANOVA including within and between factors was used for the comparison of body weight, food intake, fasting blood glucose, GTT, MWC, and MIR within and between groups pre, during, and the post-experimental period. When needed, Tukey’s *post hoc* analyses were deployed. Skeletal muscle morphology, redox balance and inflammatory status between groups were compared using one-way ANOVAs with Tukey’s *post hoc* comparisons. A Pearson’s correlation coefficient analysis was used to analyze the associations between fasting blood glucose and GTT with the neuromuscular parameters, redox balance, and inflammatory status. Statistical significance was accepted with *p* < 0.05. All statistical analyses were performed using the GraphPad Prism 6.0 software (GraphPad Software, Inc., CA, United States).

## Results

**FIGURE 3:**
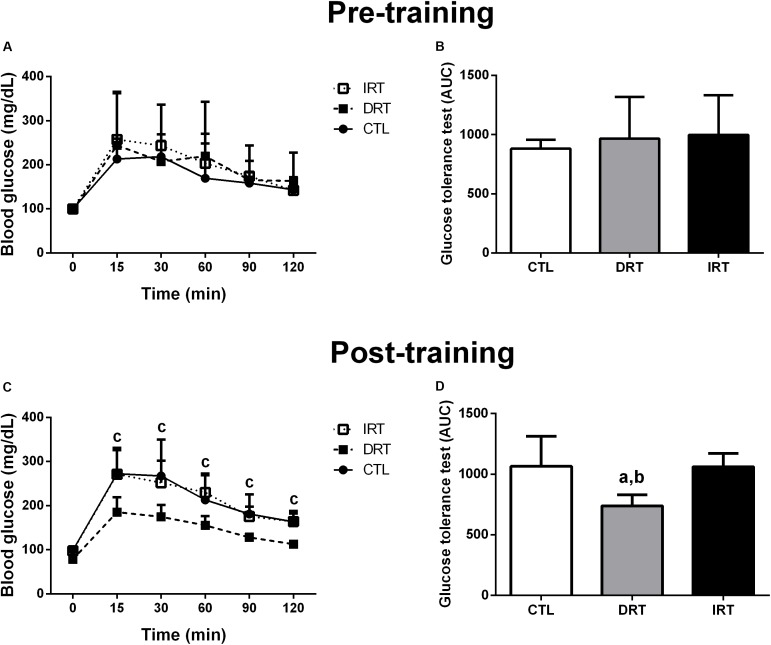
Glucose tolerance test pre-, and post-training. Data are presented as mean ± SD. Glucose tolerance test pre- **(A)** and post-training **(C)**, behavior of the area under the curve of the glucose tolerance test pre- **(B)** and post-training **(D)**. CTL, control group; DRT, dynamic resistance training; IRT, isometric resistance training; AUC, area under the curve of glycaemia behavior during GTT. A repeated measure two-way ANOVA period followed by the Tukey’s *post hoc* was adopted to compare glycaemia behavior during the glucose tolerance test within and between groups **(A,C)**. The one-way ANOVA followed by the Tukey’s *post hoc* test was adopted to verify the difference in the area under the curve glucose tolerance test between groups **(B,D)**. ^a^*p* = <0.01115 vs. CTL (between groups); ^b^*p* = <0.01115 vs. IRT group (between groups); ^c^*p* = 0.0111 vs. Time 0 min (within group), (*n* = 5 rats per group).

There were no significant interactions for time by group in body mass or food intake pre- or post-training between groups. Further, non-significant interactions were noted for fasting blood glucose and GTT pre-training between groups (*p* > 0.05). There was a group interaction for fasting blood glucose and glucose tolerance post-training (*p* = 0.0111). DRT reduced fasting blood glucose and improved glucose tolerance when compared to CTL and IRT groups. Data are shown in Table 1 and Figure 3.

**Table 1:** Body mass, food intake, and glucose analysis pre and post-training.

	CTL *N* = 5		DRT *N* = 5		IRT *N* = 5	
	Pre	Post	Pre	Post	Pre	Post
BW (g)	378 ± 20	438 ± 29	368 ± 26	428 ± 43	348 ± 40	404 ± 41
FI (g)	249 ± 20	305 ± 27	246 ± 43	315 ± 28	241 ± 46	313 ± 12
FBG (mg/dL)	100 ± 4	99 ± 4	97 ± 6	79 ± 4^a,b,c^	100 ± 7	98 ± 4
GTT (AUC)	882 ± 76	1066 ± 247	967 ± 352	740 ± 89^a,b,c^	1000 ± 333	1061 ± 112

*Data are presented as mean ± SD. CTL, control group; DRT, dynamic resistance training; IRT, isometric resistance training; BW, body weight; FI, food intake; FBG, fasting blood glucose; GTT, glucose tolerance test; AUC, area under the curve of glycaemia behavior during GTT. A repeated measure two-way ANOVA followed by the Tukey’s post hoc was adopted to verify the difference of the variables within and between groups. ^a^p = <0.01115 vs. Pre (within group); ^b^p = <0.01115 vs. CTL group (between groups); ^c^p = <0.01115 vs. IRT group (between groups).*

**FIGURE 4:**
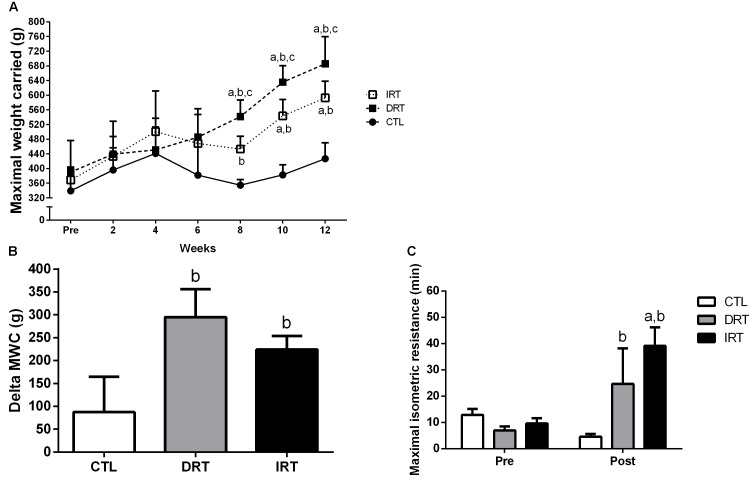
Data are presented as mean ± SD. **(A)** Maximal weight carried test; **(B)** Delta of the maximal weight carried test; **(C)** Maximal isometric resistance. CTL, control group; DRT, dynamic resistance training; IRT, isometric resistance training; MWC, maximal weight carried. A repeated measures two-way ANOVA including within and between groups analysis followed by the Tukey’s *post hoc* test was adopted to compare the behavior of muscular strength pre, during and the post-experimental period **(A)** and maximal isometric resistance pre- and post-training **(C)**. The one-way ANOVA followed by the Tukey’s *post hoc* test was adopted to compare the delta maximal weight carried test between groups **(B)**. For panel **A**: ^a^*p* < 0.0001 vs. Pre (within group); ^b^*p* < 0.0001 vs. CTL group (between group); ^c^*p* < 0.0001 vs. IRT group (between group). For panel **B**: ^b^*p* = 0.0004 vs. CTL group. For panel **C**: ^a^*p* = 0.0338 vs. Pre (within group); ^b^*p* = 0.0055 vs. CTL group (between groups), (*n* = 5 rats per group).

There were no differences in muscle strength between groups at baseline (339 ± 65, 390 ± 86, and 369 ± 31 g for CTL, DRT, and IRT, respectively, *p >* 0.05). The significant interaction for time and group yielding the findings that DRT (686 ± 66 g) and IRT (593 ± 41 g) groups showed increased muscle strength at post-test compared to baseline (*p* < 0.0001). Follow up analyses revealed greater muscle strength in the DRT group as compared with CTL and IRT groups at the eighth (542 ± 41, 355 ± 13, and 453 ± 31 g for DRT, CTL, and IRT, respectively; *p* < 0.0001), tenth (636 ± 40, 383 ± 24, and 544 ± 40 g for DRT, CTL, and IRT, respectively; *p* < 0.0001), and twelfth weeks (*p* < 0.0001), respectively. In addition, the strength gain (delta; i.e., post – pre-training) was similar between the DRT (295 ± 54 g) and IRT (224 ± 27 g; *p* > 0.05) groups, but different from the CTL (88 ± 69 g; *p* = 0.0004) group. Baseline values of MIR did not differ between groups (13 ± 5, 7 ± 3, and 10 ± 5 min for CTL, DRT, and IRT, respectively, *p* > 0.05). However, there was significant group × time interaction where DRT (25 ± 25 min) and IRT (39 ± 40 min) groups increased MIR after training compared to the CTL group (5 ± 5 min; *p* = 0.0055). In addition, the IRT group increase MIR compared baseline data (*p* = 0.0338). Data presented in Figure 4.

**FIGURE 5:**
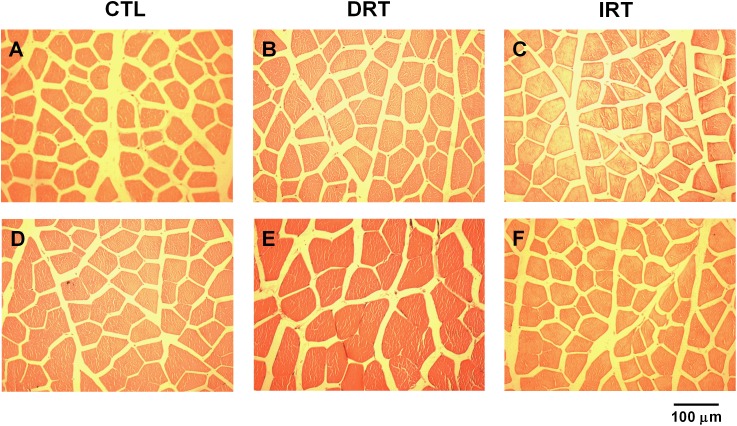
Representative photomicrographs of muscle sections. Hematoxylin-eosing staining. Panels **A**–**C** present the cross-sectional area of the tibialis anterior muscle. Panels **D**–**F** present the cross-sectional area of the quadriceps muscle. Magnification 200×, (*n* = 5 rats per group).

The weight of the tibialis anterior and quadriceps muscles were similar between groups at the end of the training (*p* > 0.05). The DRT elicited more hypertrophy in the tibialis anterior and quadriceps muscles as compared to the CTL and IRT groups (*p <* 0.0001). Data are shown in Table 2 and the representative photomicrograph in Figure 5.

**Table 2:** Weight and muscular morphometry after 12 weeks of training.

Skeletal Muscle Morphology	CTL *N* = 5	DRT *N* = 5	IRT *N* = 5
TAW (mg)	768 ± 77	792 ± 119	664 ± 34
QW (mg)	3.100 ± 404	3.238 ± 319	2.664 ± 205
CSATA (μm^2^)	951 ± 114	2709 ± 589^a,b^	1145 ± 232
CSAQ (μm^2^)	1187 ± 214	2929 ± 312^a,b^	1433 ± 222

*Data are presented as mean ± SD. CTL, control group; DRT, dynamic resistance training; IRT, isometric resistance training; TAW, tibialis anterior weight; QW, quadriceps weight; CSATA, cross-sectional area of tibialis anterior; CSAQ, cross-sectional area of quadriceps. The one-way ANOVA followed by the Tukey’s post hoc test was adopted to verify the difference in the muscular weights and hypertrophy between groups. ^a^p < 0.0001 vs. CTL group (between groups); ^b^p < 0.0001 vs. IRT group (between groups).*

**FIGURE 6:**
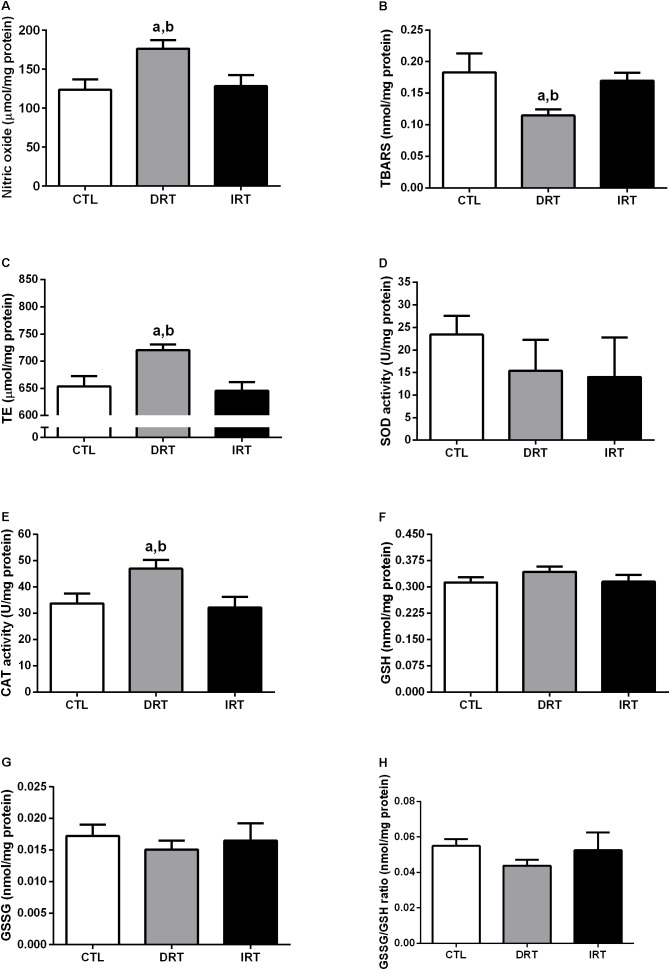
Muscular redox balance. Data are presented as mean ± SD. Nitric oxide **(A)**, thiobarbituric acid reactive substances **(B)**, trolox equivalent **(C)**, superoxide dismutase **(D)**, catalase **(E)**, glutathione **(F)**, glutathione disulfide **(G)**, and glutathione disulfide/glutathione ratio **(H)**. CTL, control group; DRT, dynamic resistance training; IRT, isometric resistance training; TBARS, thiobarbituric acid reactive substances; TE, trolox equivalent; SOD, superoxide dismutase; CAT, catalase; GSH, glutathione; GSSG, glutathione disulfide. The one-way ANOVA followed by the Tukey’s *post-hoc* test was adopted to verify the difference between the groups. ^a^*p* < 0.0001 vs. CTL group (between groups); ^b^*p* < 0.0001 vs. IRT group (between groups), (*n* = 5 rats per group).

After 12 weeks of DRT there was an increase in NO (176 ± 11, 124 ± 13, and 128 ± 14 μmol/mg of protein for DRT, CTL, and IRT groups, respectively; *p* < 0.0001), trolox equivalent (721 ± 10, 654 ± 19, and 646 ± 16 μmol/mg of protein for DRT, CTL, and IRT groups, respectively; *p* < 0.0001), catalase activity (47 ± 3, 34 ± 4, and 32 ± 4 U/mg of protein for DRT, CTL, and IRT groups, respectively; *p* < 0.0001), and a reduction of lipid peroxidation (DRT 0.11 ± 0.01 nmol/mg of protein) compared to CTL (0.18 ± 0.03 nmol/mg of protein) and IRT (0.17 ± 0.01 nmol/mg of protein) groups (*p* < 0.0001). There was no difference between groups for SOD activity (23 ± 4, 15 ± 7, and 14 ± 9 U/mg of protein for DRT, CTL, and IRT groups, respectively) GSH (0.31 ± 0.02, 0.34 ± 0.01, and 0.32 ± 0.02 nmol/mg of protein for DRT, CTL, and IRT groups, respectively), GSSG (0.017 ± 0.002, 0.015 ± 0.001, and 0.016 ± 0.003 nmol/mg of protein for DRT, CTL, and IRT groups, respectively) and GSSG/GSH ratio (0.055 ± 0.004, 0.044 ± 0.003, and 0.053 ± 0.010 nmol/mg of protein for DRT, CTL, and IRT groups, respectively) (*p* > 0.05). Data are presented in Figure 6.

**FIGURE 7:**
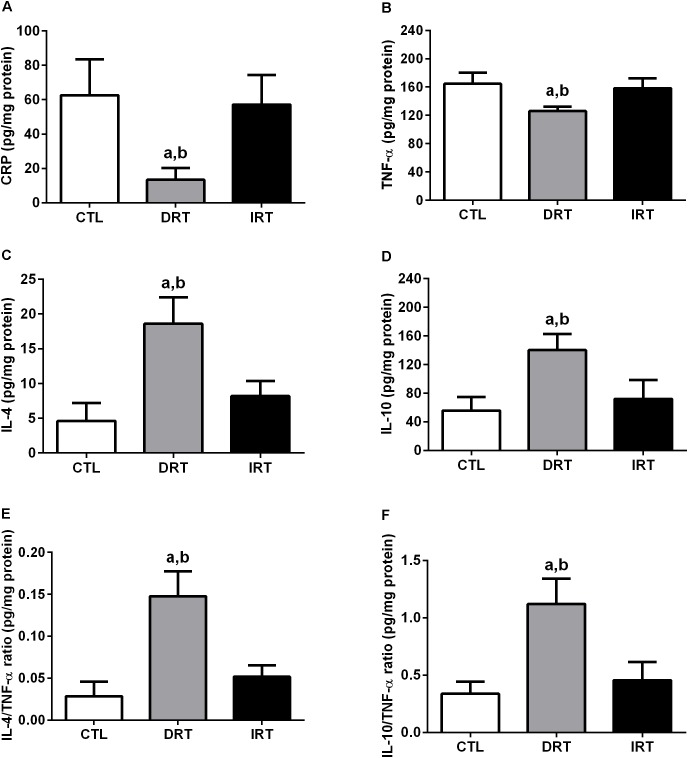
Muscular inflammatory states. Data are presented as mean ± SD. C-reactive protein **(A)**, tumor necrosis factor alpha **(B)**, interleukin-4 **(C)**, interleukin-10 (**D**), interleukin-4/tumor necrosis factor alpha ratio **(E)** and interleukin-10/tumor necrosis factor alpha ratio **(F)**. CTL, control group; DRT, dynamic resistance training; IRT, isometric resistance training; CRP, c-reactive protein; TNF-α: tumor necrosis factor alpha; IL-4, interleukin-4; IL-10, interleukin-10. The one-way ANOVA followed by the Tukey’s *post-hoc* test was adopted to verify the difference between the groups. ^a^*p* < 0.0001 vs. CTL group (between groups); ^b^*p* < 0.0001 vs. IRT group (between groups), (*n* = 5 rats per group).

There was a decrease in CRP (13 ± 7, 63 ± 21, and 57 ± 17 pg/mg of protein for DRT, CTL, and IRT groups, respectively; *p* = 0.0007) and TNF-α (126 ± 6, 165 ± 16, and 158 ± 14 pg/mg of protein for DRT, CTL, and IRT groups, respectively; *p* = 0.0009) levels in the DRT group as compared with CTL and IRT groups. There was an increase in IL-4 (19 ± 4, 5 ± 3, and 8 ± 2 pg/mg of protein for DRT, CTL, and IRT groups, respectively; *p* < 0.0001), IL-10 (140 ± 22, 55 ± 19, and 72 ± 26 pg/mg of protein for DRT, CTL, and IRT groups, respectively; *p* = 0.0002), IL-4/TNF-α ratio (0.15 ± 0.03, 0.03 ± 0.02, and 0.05 ± 0.01 pg/mg of protein for DRT, CTL, and IRT groups, respectively; *p* < 0.0001), and IL-10/TNF-α ratio (1.12 ± 0.22, 0.34 ± 0.10, and 0.46 ± 0.16 pg/mg of protein for DRT, CTL, and IRT groups, respectively; *p* < 0.0001) in the DRT as compared with CTL and IRT groups. Data are shown in Figure 7.

Pearson’s correlation tests revealed an association between fasting glucose with MWC, MIR, CSA; tibialis anterior and quadriceps, NO, TBARS, Trolox equivalent, CAT, GSH, GSSG, GSSG /GSH ratio, CRP, TNF-α, IL-4, IL-10, IL-4/TNF-α ratio and IL-10/TNF-α ratio. However, these variables were not associated with GTT (*p* > 0.05). Values are shown in Table 3.

**Table 3:** Pearson’s correlation between studied variables.

	FBG			GTT		
Variables	*r*	*R*^2^	*P*	*r*	*R*^2^	*p*
**Neuromuscular parameters**						
MWC	-0.65	0.42	**<0.0001**	-0.09	0.00	0.6318
MIR	-0.29	0.08	0.1095	0.01	0.00	0.9429
CSATA	-0.94	0.89	**<0.0001**	0.00	0.00	0.9753
CSAQ	-0.90	0.81	**<0.0001**	0.07	0.00	0.7915
**Redox balance**						
NO	-0.76	0.58	**0.0009**	0.03	0.00	0.8898
TBARS	0.75	0.56	**0.0013**	-0.14	0.02	0.6077
TE	-0.79	0.63	**0.0004**	-0.09	0.00	0.7475
SOD	0.11	0.01	0.6860	0.03	0.00	0.9147
CAT	-0.85	0.72	**<0.0001**	-0.026	0.07	0.3406
GSH	-0.64	0.41	**0.0096**	-0.38	0.14	0.1539
GSSG	0.57	0.33	**0.0242**	0.23	0.05	0.4082
GSSG/GSH ratio	0.74	0.55	**0.0013**	0.36	0.13	0.1786
**Inflammatory status**						
CRP	0.74	0.54	**0.0016**	0.20	0.04	0.4630
TNF-α	0.78	0.61	**0.0005**	-0.05	0.00	0.8412
IL-4	-0.82	0.68	**0.0001**	-0.06	0.00	0.8075
IL-10	-0.86	0.75	**<0.0001**	-0.19	0.03	0.4967
IL-4/TNF-α ratio	-0.86	0.74	**<0.0001**	-0.04	0.00	0.8763
IL-10/TNF-α ratio	-0.90	0.82	**<0.0001**	-0.08	0.00	0.7627

*FBG, fasting blood glucose; GTT, glucose tolerance test; MWC, maximal weight carried; MIR, maximal isometric resistance; CSATA, cross-sectional area of tibialis anterior; CSAQ, cross-sectional area of quadriceps; NO, nitric oxide; TBARS, thiobarbituric acid reactive substances; TE, trolox equivalent; SOD, superoxide dismutase; CAT, catalase; GSH, glutathione; GSSG, glutathione disulfide; CRP, c-reactive protein; TNF-α, tumor necrosis factor alpha; IL-4, interleukin-4; IL-10, interleukin-10. The values in bold highlight the correlations that were significant.*

## Discussion

Dynamic resistance training is widely recognized as an important tool to promote glycemic homeostasis due to increased muscle mass, improved redox and inflammatory status (Church et al., 2010; Nunes et al., 2016; Maleki and Tartibian, 2018). However, these effects are less studied with IRT despite its wide implementation in rehabilitative settings. Thus, the main purpose of this study was to compare the effects of DRT versus IRT on fasting glucose, glucose tolerance, muscle redox and inflammatory states, and muscle strength and hypertrophy in rats. Results revealed that IRT was unable to match the improvements in glycemic homeostasis compared to the DRT, likely explained by the small changes in muscle function and absence of hypertrophy, redox, and inflammatory muscle profiles following IRT.

Isometric resistance training reduced glucose intolerance in obese mice induced by changes in diet along with increased GLUT-4 in the rectus femoris, soleus, and gastrocnemius muscles. These adaptations together may explain the improvement in glucose tolerance in obese mice (Kruger et al., 2013). It is likely that this absence of improved glycemic homeostasis following IRT could be explained by the intensity used and failure to evoke similar muscular adaptations as observed in the DRT. Moreover, rats in the DRT group climbed the ladder uninterrupted for 1 min, which may have generated a higher energy expenditure compared to the IRT. Thus, successive sessions of DRT increased the caloric expenditure and these adjustments are possibly involved in the reduction of fasting glucose and increased glucose tolerance. As such, the total work between groups was different probably because of the displacement in the DRT, despite the same intensity and duration of training. Kruger et al. (2013) found that higher intensity IRT was able to decrease glucose intolerance, such intensity is due to excess body fat, around 20% relative to lean mice (Rosa et al., 2015). Obese mice in the study by Kruger et al. (2013) performed the isometric exercise at an angulation (i.e., 90° position relative to the ground) that requires greater effort of the animal to remain in the static position, while in our study the animals performed the exercise with the apparatus at 80°, which biomechanically gives less effort. Supporting the recommendation for higher intensity, it has been noted that glucose uptake occurs in an intensity-dependent manner in rats and mice (Ihlemann et al., 1999; Chambers et al., 2009). In addition, Kido et al. (2016) applied electrical stimuli to the *in situ* gastrocnemius muscle of rats with the aim of producing maximal isometric contractions and observed an increase in GLUT-4 translocation via IGF-1 signaling. Taken together, these data would support that the intensity of IRT seems to partially influence the adaptations in glucose metabolism.

An additional factor to be considered is the initial clinical condition of the rat. The sample studied by Kruger et al. (2013) observed improvements in glucose tolerance in obese mice whereas the current sample was considered normal weight. The pathophysiological changes generated by the alteration in insulin metabolism in obese rats seem to increase sensitivity to exercise training (Shekarchizadeh Esfahani et al., 2013), which may explain the efficacy of IRT on glucose metabolism in obese mice, which did not occur in the present investigation in lean rats. Thus, indicating a ceiling effect on glucose metabolism in normoweight rats.

We observed that DRT improved glycaemia, increased muscle strength and CSA. The reduction in fasting glycaemia and improvement in postprandial glucose tolerance may, due to the muscle hypertrophy. These results occurred without changes in body mass, muscles weight, or food consumption. This reinforces the importance of hypertrophy to improve glucose metabolism (Miller et al., 1984; Lee et al., 2012; Tang et al., 2014). To note, our results revealed that CSA of the muscles was inversely correlated with fasting blood glucose values, supporting the notion that DRT may be a covariate for this negative association.

Other studies with rodents demonstrated that IRT generated muscle hypertrophy and strength when protocols were progressively intensified and included an angulation (e.g., 90°) (Adams et al., 2004; Mounier et al., 2007; Kruger et al., 2013).

This reflection corroborates our findings, where higher levels of muscle strength seem to protect against increased blood glucose in the DRT group. Despite the same gain in muscle strength, IRT did not cause changes in glycemic homeostasis. This finding may suggest that other factors may be involved, particularly, increased CSA.

In some clinical conditions, such as obesity, diabetes, hypertension, endothelial dysfunction and chronic kidney disease, DRT and IRT were found to be satisfactory to improve glycemic control, reduce oxidative stress, and inflammation (Figard et al., 2006; Panveloski-Costa et al., 2011; Kruger et al., 2013; Talebi-Garakani and Safarzade, 2013; Neves et al., 2016; Souza M.K. et al., 2018). The authors were unable to find previous studies that compared the impact of both training models (dynamic vs. static) on glycemic homeostasis, more specifically, the influence of IRT on inflammation, redox status and hypertrophy. Thus, our research adds important findings to literature and reveals that the intensity and volume IRT protocol was not sufficient to induce changes in the above mentioned parameters.

DRT improved oxidative biomarkers in rats, as demonstrated by the increased bioavailability of NO, reduced TBARS, improved total antioxidant defense and increased catalase activity, which promote glycemic function. Indeed, it is indicated that the transient increase in the production of reactive oxygen species (ROS) during an exercise session induces a long-term improvement in the antioxidant system and in insulin sensitivity, with a concomitant increase in glucose uptake in both trained and untrained healthy men. Thus, our results show that the improved glycemic homeostasis was associated with a reduction of pro-oxidant markers and increase in antioxidant defense following DRT. Therefore, increasing ROS during acute exercise may be important to reduce the risk of metabolic diseases following a training period (Ristow et al., 2009).

The IRT prescribed in the present study did not promote improvements in antioxidant defense or glucose uptake, possibly because the training load at 30% MWC was insufficient. Peters et al. (2006) demonstrated that a session of isometric resistance exercise increased ROS after a load of 50% maximal voluntary isometric contraction (MVIC) in subjects with hypertension, and long-term reduced the production of ROS and improved the antioxidant defense. Thus, these data indicate that higher-intensity and longer training duration are important to improve adaptation in the redox balance if implementing IRT (Peters et al., 2006).

The exogenous administration of antioxidant N-acetyl-L-cysteine during muscle contraction impairs the glucose uptake by myocytes by decreasing the expression of ROS, thus demonstrating the importance of free radicals produced acutely to generate glycemic benefits (Merry et al., 2009). However, these inferences should be taken with caution, since the studies cited above were with humans, and we did not analyze the acute expression of ROS.

In addition, it is indicated that the glucose uptake by muscle contraction occurs by several mechanisms not fully understood (Sylow et al., 2017), and one of the signs involved in the regulation of glucose uptake through muscle contraction involves the participation of NO (McConell et al., 2012). In this sense, the lack of modification in glycemic homeostasis observed in IRT may be associated with the lack of increase in NO bioavailability. In fact, administration of NG-monomethyl-L-arginine (L-NMMA), a non-selective inhibitor of NOS (e.g., enzymes that produce NO) induced a decrease of glucose uptake in healthy and participants with type 2 diabetes (Bradley et al., 1999; Kingwell et al., 2002). In contrast, DRT was efficient to increase NO, and consequently glycemic homeostasis. One of the proposed mechanisms suggests that NO has a key role in the stimulation of the GLUT-4 translocation to the myocyte membrane (McConell et al., 2012). Thus, DRT compared to IRT improves glycemic homeostasis and improvement in the mechanisms involved in pro- and antioxidant systems, consequently reducing oxidative stress and inflammation.

Oxidative stress is associated with instigating insulin resistance by up-regulating the production of pro-inflammatory cytokines, such as CRP and TNF-α, which induce damage to proteins in the insulin cascade (Styskal et al., 2012; Xie et al., 2017). Previous evidence has shown that DRT in rats with diabetes (Talebi-Garakani and Safarzade, 2013), middle-aged (Jung et al., 2015) and obesity induced by a hyperlipidic diet, reduced pro-inflammatory markers, and was associated with improved insulin sensitivity in skeletal muscle (Panveloski-Costa et al., 2011). The impact of IRT on the modulation of the inflammatory profile and its influence on glucose metabolism was not evaluated in the current scientific literature. Although speculative, we anticipate that glycemic parameters did not improve after IRT due to, in part, the lack of modification in the inflammatory profile. In contrast, the observed data from the DRT group corroborates with the studies mentioned above, since it induced a decrease in the expression of pro-inflammatory cytokines (CRP and TNF-α), increased anti-inflammatory (IL-4 and IL-10), and improved the inflammatory balance (e.g., increased the IL-4/TNF-α and IL-10/TNF-α ratios). We demonstrated that the DRT benefits the metabolism of glucose in the myocytes by mechanisms involving the reduction of inflammation, as pro-inflammatory cytokines such as CRP and TNF-α promote the worsening of glucose uptake through peripheral resistance in insulin action (D’Alessandris et al., 2007; Xie et al., 2017).

The current data also supports the negative relationship between muscle strength, muscle hypertrophy, NO, total antioxidant capacity, catalase activity, GSH, IL-4, IL-10, IL-4/TNF-α ratio, and IL-10/TNF ratio with fasting glycaemia. Moreover, TBARS, GSSG, GSSG/GSH rate, CRP, and TNF-α were positively associated with increased blood glucose. Nevertheless, it is worth mentioning that the DRT altered the distribution of the values found, allowing a viable dispersion to obtain the correlation. The present study reported mechanisms that directly influence glucose metabolism in muscle cells, and add some evidence that IRT did not cause changes in glycemic homeostasis, at least in part to the lack of modification of inflammatory and redox parameters and muscle CSA. This indicates that the prescribed intensity and volume were not potent enough to cause metabolic changes and hypertrophy. We way speculate that the DRT was more effective due to the higher caloric expenditure and the dynamic training component. Unfortunately, the caloric expenditure was not measured in the current investigation.

### Limitations, Strength, and Practical Applications

Despite dedicated efforts to control extraneous influences on the investigation, this study has some limitations: (i) the analysis of the insulin resistance test would provide information about whether the improvement in glucose uptake observed in the DRT was due to an improvement in glucose tolerance or associated with an increase in insulin sensitivity. DRT improved both fasting glycaemia and GTT by improving glycemic homeostasis; (ii) measurement of inducible nitric oxide synthase (iNOS) protein expression and s-nitrosylation of the insulin cascade (IR-β, IRS-1 and AKT) in muscle tissue would provide additional elements for understanding the lack of modification in the glycemic homeostasis observed in the IRT group, and the role of DRT in decreasing inflammation and improving blood glucose. However, it is worth noting that the current investigation evaluated the redox and inflammatory balance in the skeletal muscle indicating that DRT likely reduced s-nitrosylation of the insulin cascade, since it increased NO bioavailability, improved antioxidant defense, and the expression of anti-inflammatory cytokines in the muscle, which was not found in IRT; (iii) our findings demonstrated that IRT did not alter the redox and inflammatory balance of muscle and, therefore, the evaluation of NF-κB pathway in the myocytes could provide relevant information for the non-response in glycemic homeostasis; (iv) this present study was limited by a small sample size in each group (*n* = 5), which may have restricted our capacity to reveal other statistical differences. In addition, all the animals used in the study were from the same littermate, thus reducing generalizability of the results (Kilkenny et al., 2010; Holmdahl and Malissen, 2012).

As mentioned previously, the training intensity chosen for the present study was based on findings from a pilot study wherein animals held loads ranging from 5 to 70% of their maximal load for 60 s. Findings for the rats were similar to those implemented in humans, where resistance average 30% of maximal load (Millar et al., 2014; Souza L.R. et al., 2018). As such, the current protocol may serve as a translational piece between rodent and human subjects as the resistance and maximal loads tests are similar in both cohorts. Thus, this study renders a perspective to test the chronic influence of IRT on metabolic settings in human models. Future studies may consider imposing different IRT intensities and volumes, in order to verify their influence on the NF-κB pathway in glycemic homeostasis.

## Conclusion

In summary, we concluded that IRT did not modify glycemic homeostasis, since it did not generate muscle hypertrophy, nor alter the redox or inflammatory profile. Alternatively, DRT caused favorable inflammatory and redox changes in the muscular tissue. Although muscle strength improvement was similar between the DRT and IRT groups. At the end of the training MIR increased in both DRT and IRT compared to CTL, however, in relation to pre-training increased only in the IRT. Future studies are needed to test a greater volume of IRT, or to prescribe the intensity in a progressive way in order to verify if such parameters can improve glycemic homeostasis in the time-course against sedentary lifestyle.

## Ethics Statement

The present study provide all the listed requirements.

## Author Contributions

RN, TR, MS, AO, GG, BB, and LD performed the experiments. RN, TR, and MS analyzed the data. RN, TR, and MS drafted the manuscript. RN, TR, MS, AO, GG, BB, LS, LD, HS, WS, JP, and MM edited and revised the manuscript. RN, TR, MS, AO, GG, BB, LS, LD, HS, WS, JP, and MM approved final version of the manuscript.

## Conflict of Interest Statement

The authors declare that the research was conducted in the absence of any commercial or financial relationships that could be construed as a potential conflict of interest.
